# Sensitivity and Predictive Value of the Frozen Section of Sentinel Lymph Node Biopsy in the Post-neoadjuvant Setting: Experience From a Tertiary Care Hospital in a Resource-Limited Country

**DOI:** 10.7759/cureus.72412

**Published:** 2024-10-26

**Authors:** Fatima Safdar, Lubna Vohra, Romana Idress

**Affiliations:** 1 Histopathology, Pathology and Laboratory Medicine, Aga Khan University Hospital, Karachi, PAK; 2 Surgery, Aga Khan University Hospital, Karachi, PAK

**Keywords:** axillary dissection, breast cancer, frozen biopsy, neoadjuvant chemotherapy, sentinel lymph node biopsy

## Abstract

Background

Axillary lymph node status is one of the most important prognostic factors in breast cancer treatment, which can be confirmed by sentinel lymph node biopsy (SLNB). Intraoperative frozen section is an alternative method for SLNB, which can reduce the risks associated with secondary surgery. The feasibility and accuracy of SLNB after post-neoadjuvant chemotherapy (NACT) are affected by many factors as lymphatic drainage from the breast could be impaired due to fibrosis, fat necrosis, and granulation tissue formation, thus hampering the detection of the sentinel lymph node and afterward interpretation by pathologists due to therapy-related changes. Despite the increasing use of SLNB in post-NACT settings, there is still limited information on the accuracy of SLNB in resource-limited countries.

Objective

Our study aims to detect the sensitivity and predictive value of frozen section SLNB in the post-NACT setting while comparing it with final permanent histopathological results and considering final permanent histopathological results as standard.

Materials and methods

A total of 286 patients meeting the inclusion criteria from 2021 to 2022 were included in the study. Hematoxylin and eosin (H&E)-stained microscopic glass slides of frozen SLNB after NACT, permanent paraffin-embedded sections, and immunohistochemical stains were retrieved and reviewed. For all the categorical variables, including histologic type and grade, frequencies and percentages were obtained. Measures of central tendency and variability for continuous data such as age, number of sentinel lymph nodes received, and size of the largest nodal deposit were calculated. The chi-square test was used for the comparison of qualitative variables. A p-value of less than or equal to 0.05 was considered statistically significant.

Results

The median age of presentation was 47 years (range = 39 to 55 years). The median number of sentinel lymph nodes received was three (range = 2-4). At the time of frozen section reporting, out of a total of 286 cases, 229 (80.1%) cases were labeled as negative, 55 (19.2%) cases as positive, and two (0.7%) cases were deferred for permanent section results. Out of 229 cases labeled as negative at the time of the frozen section, 220 (76.9%) cases were true negative confirmed on permanent sections. A total of 66 (33.1%) cases were true positive, including two deferred cases and nine false negative cases, in addition to 55 cases labeled as positive on the initial frozen section. The study showed sensitivity, specificity, and accuracy of frozen section analysis of SLNB at 83.00%, 100%, and 96.15%, respectively, with a false negative rate (FNR) rate of 16.7%.

Conclusion

Further follow-up studies to definitively determine the role of SLNB following post-NACT in patients who did not undergo axillary lymph node dissection (ALND) are needed. Continuous monitoring of the rate of false positives and false negatives of frozen sections on SLNB is essential as feedback for pathologists.

## Introduction

Axillary lymph node status is an important prognostic factor in breast cancer and is used to guide further treatment plans [1,2]. Neo-adjuvant chemotherapy (NACT) is given to patients with advanced disease or early breast cancer, with the aim of downstaging and improving the likelihood of breast-conserving surgery [3,4]. The status of axillary involvement after chemotherapy is significant; however, removing all nodes for residual nodal disease assessment subjects patients to morbidity, and only a small percentage of patients will benefit from surgery. To avoid the complications associated with axillary lymph node dissection (ALND) such as lymphedema, upper extremity sensory and motor deficits, and alteration in shoulder mobility, it is preferable to proceed with the less invasive sentinel lymph node biopsy (SLNB) [3-6]. A sentinel lymph node is defined as the first draining lymph node that receives lymph flow from the primary tumor and is most likely to have tumor cells [7]. Intraoperative frozen section is an alternative method for SLNB, which can reduce the risks associated with secondary surgery. A delayed second surgical procedure is avoided when SLNB metastases are found on the frozen section; instead, ALND can be carried out immediately. For this reason, most institutions follow the frozen section as a routine procedure [5,8-10].

The role and timing of ALND for patients who have received NACT are challenging [8]. Reliable data for the detection rate, accuracy, false negative rate (FNR), and the number of regional relapses are available when a biopsy is performed before NACT; however, in the context of NACT, limited data are available. The feasibility and accuracy of SLNB after NACT are affected by many factors as lymphatic drainage from the breast could be impaired, thus hampering the detection of the sentinel lymph node [3]. NACT causes fibrosis, fat necrosis, and granulation tissue formation, which alters lymphatic drainage patterns. Tumor regression in the axilla can also follow a non-uniform pattern, leading to inaccurate findings. The interpretation can be challenging at times by pathologists due to the presence of extensive therapy-related changes and fat replacement. The FNR of the SLNB frozen section result reported in the literature ranges from 5.5 and 43% [11-13].

Despite the increasing use of SLNB in NACT settings, there is still limited information on the accuracy of SLNB in resource-limited countries. This study aims to present the data of SLNB following NACT in one of the largest tertiary care hospitals in Pakistan. Our study aims to detect the sensitivity and predictive value of frozen section SLNB in post-NACT while comparing it with final permanent histopathological results. The final permanent histopathological results were considered standard.

## Materials and methods

Study design and inclusion criteria

This a descriptive observational study. Patients older than 18 years, who were initially diagnosed with breast cancer of any histologic type and grade as per the modified Bloom-Richardson grading system, stage T1 to T4, N0 to N1 by Section of Histopathology, The Aga Khan Hospital, Karachi from 2021 to 2022 and subsequently received NACT, were included in the study. Poorly preserved biopsy material, which was insufficient to assess all the histological features, was excluded. All the cases meeting the inclusion criteria from 2021 to 2022 were searched electronically in the institutional Integrated Laboratory Management System (ILMS).

Data collection

The study protocol was approved as an exemption by the Institutional Ethical Review Committee (ERC number: 2022-7303-20844), as the study was conducted by reviewing archived material, and no identifiable patient information was included. Informed verbal consent was obtained from each patient via telephone. Patient information such as demographics, including age, histologic type, grade, number of sentinel lymph nodes received, size of the largest nodal deposit, and permanent frozen section results were extracted from the histopathology reports. Hematoxylin and eosin (H&E)-stained microscopic glass slides of frozen SLNB after NACT, permanent paraffin-embedded sections, and immunohistochemical stains were retrieved and reviewed by a senior pathologist with experience in breast pathology. All the sentinel lymph nodes received for frozen evaluation had been routinely grossed and subsequently formalin-fixed according to the guidelines given in the Manual of Surgical Pathology [14]. Routinely four levels are done and examined on every frozen SLNB sample reported in our setting. The frozen and permanent formalin-fixed, paraffin-embedded tissue sections were stained with H&E. Immunohistochemical stain cytokeratin AE1/AE3 was performed on permanent paraffin-embedded tissue to confirm nodal deposit on all cases of invasive lobular carcinoma and sentinel lymph nodes exhibiting extensive therapy-related changes. Immunohistochemical stain cytokeratin AE1/AE3 has not been performed on every permanent paraffin-embedded section due to limited financial budgets. The tumor focus of any size in frozen and permanent sections of SLNB was considered positive and was recorded. The results of frozen and permanent sections of SLNB were compared to evaluate sensitivity, specificity, and FNR while considering permanent paraffin-embedded sections as standard. The number and type of discrepancies, including sampling and interpretation errors, were noted. The number of deferred cases and causes for deferral were also evaluated. The deferred cases were considered false negative cases during the statistical analysis.

The study has also been registered in the ClinicalTrials.gov registry (NCT06401590) as it will broaden the national database regarding predictive values and FNR of SLNB in post-NACT settings.

Statistical analysis

Stata version 17 (StataCorp LLC, College Station, TX) was used to conduct descriptive statistical analysis. For all the categorical variables, including histologic type and grade, frequencies and percentages were obtained. Measures of central tendency and variability for continuous data such as age, number of sentinel lymph nodes received, and size of the largest nodal deposit were calculated. The chi-square test was used for comparison of qualitative variables. A p-value of less or equal to 0.05 was considered statistically significant. Manual and computerized validity checks for the data were performed to ensure reliability and to avoid duplication of the data.

## Results

A total of 286 patients were included in the study. The median age of presentation was 47 years (range = 39 to 55 years). A total of 269 (94.1%) patients were diagnosed with invasive ductal carcinoma, 10 (3.5%) with invasive lobular carcinoma, and seven (2.4%) with metaplastic carcinoma. Out of 286 cases, 158 (55.2%) were diagnosed as grade 3, 120 (42%) as grade 2, and eight (2.8%) as grade 1, as per the modified Bloom-Richardson grading system. A total of 131/286 (45.8%) patients underwent ultrasound-guided axillary node biopsy before NACT was started, out of which 82 (28.7%) biopsies were positive for metastasis. All patients underwent SLNB after completion of their NACT. The frozen and permanent sections of these patients were diagnosed in the Section of Histopathology, Aga Khan University. The median number of sentinel lymph nodes received was three (range = 2-4). The slides of both frozen and permanent sections were reviewed by a senior pathologist with experience in breast pathology. At the time of frozen section reporting, out of a total of 286 cases, 229 (80.1%) cases were labeled as negative, 55 (19.2%) cases as positive, and two (0.7%) cases exhibited extensive fat replacement, therefore representative sections could not be assessed by pathologists. These two cases were reported as negative on limited material and were deferred to permanent sections for final diagnosis. The clinicopathological features of the patients are summarized in Table 1.

**Table 1:** Summary of demographic details and details of sentinel lymph node biopsy results on frozen and permanent sections (n = 286).

Variables	Total	Defer	Negative	Positive	Test	Test statistics	P-value
	N = 286	N = 2	N = 229	N = 55			
Patient age (years)	47 (39-55)	56.5 (48-65)	46 (38-55)	47 (39-59)	Kruskal-Wallis	Chi^2 ^(2) = 2.06	0.36
Number of sentinel lymph nodes received	3 (2-4)	4 (3-5)	3 (2-4)	3 (2-4)	Kruskal-Wallis	Chi^2 ^(2) = 4.17	0.12
Diagnosis					Chi-square	Chi^2^ (4) = 26.50	<0.001
Invasive ductal carcinoma	269 (94.1%)	1 (50.0%)	219 (95.6%)	49 (89.1%)			
Invasive lobular carcinoma	10 (3.5%)	1 (50.0%)	3 (1.3%)	6 (10.9%)			
Metaplastic carcinoma	7 (2.4%)	0 (0.0%)	7 (3.1%)	0 (0.0%)			
Histological grade before treatment					Chi-square	Chi^2^ (4) = 1.05	0.90
Grade 1	8 (2.8%)	0 (0.0%)	7 (3.1%)	1 (1.8%)			
Grade 2	120 (42.0%)	1 (50.0%)	93 (40.6%)	26 (47.3%)			
Grade 3	158 (55.2%)	1 (50.0%)	129 (56.3%)	28 (50.9%)			
Pre-treatment axillary lymph node biopsy performed					Chi-square	Chi^2^ (2) = 2.13	0.35
Not done	155 (54.2%)	1 (50.0%)	129 (56.3%)	25 (45.5%)			
Yes	131 (45.8%)	1 (50.0%)	100 (43.7%)	30 (54.5%)			
If yes, result					Chi-square	Chi^2^ (4) = 25.06	<0.001
Negative	49 (17.1%)	0 (0.0%)	48 (21.0%)	1 (1.8%)			
Positive	82 (28.7%)	1 (50.0%)	52 (22.7%)	29 (52.7%)			
Frozen section-positive largest nodal deposit size (cm)	0.6 (0.3-1.1)	-	-	0.6 (0.3-1.1)			
Sentinel lymph node biopsy (SLNB) permanent result					Chi-square	Chi^2^ (2) = 237.29	<0.001
Negative	220 (76.9%)	0 (0.0%)	220 (96.1%)	0 (0.0%)			
Positive	66 (23.1%)	2 (100%)	9 (3.9%)	55 (100%)			
Permanent positive largest nodal deposit size (cm)	0.55 (0.3-1)	0.35 (0.3-0.4)	0.2 (0.1-0.2)	0.6 (0.3-1.1)	Kruskal-Wallis	Chi^2^ (2) = 14.29	<0.001

**Figure 1:**
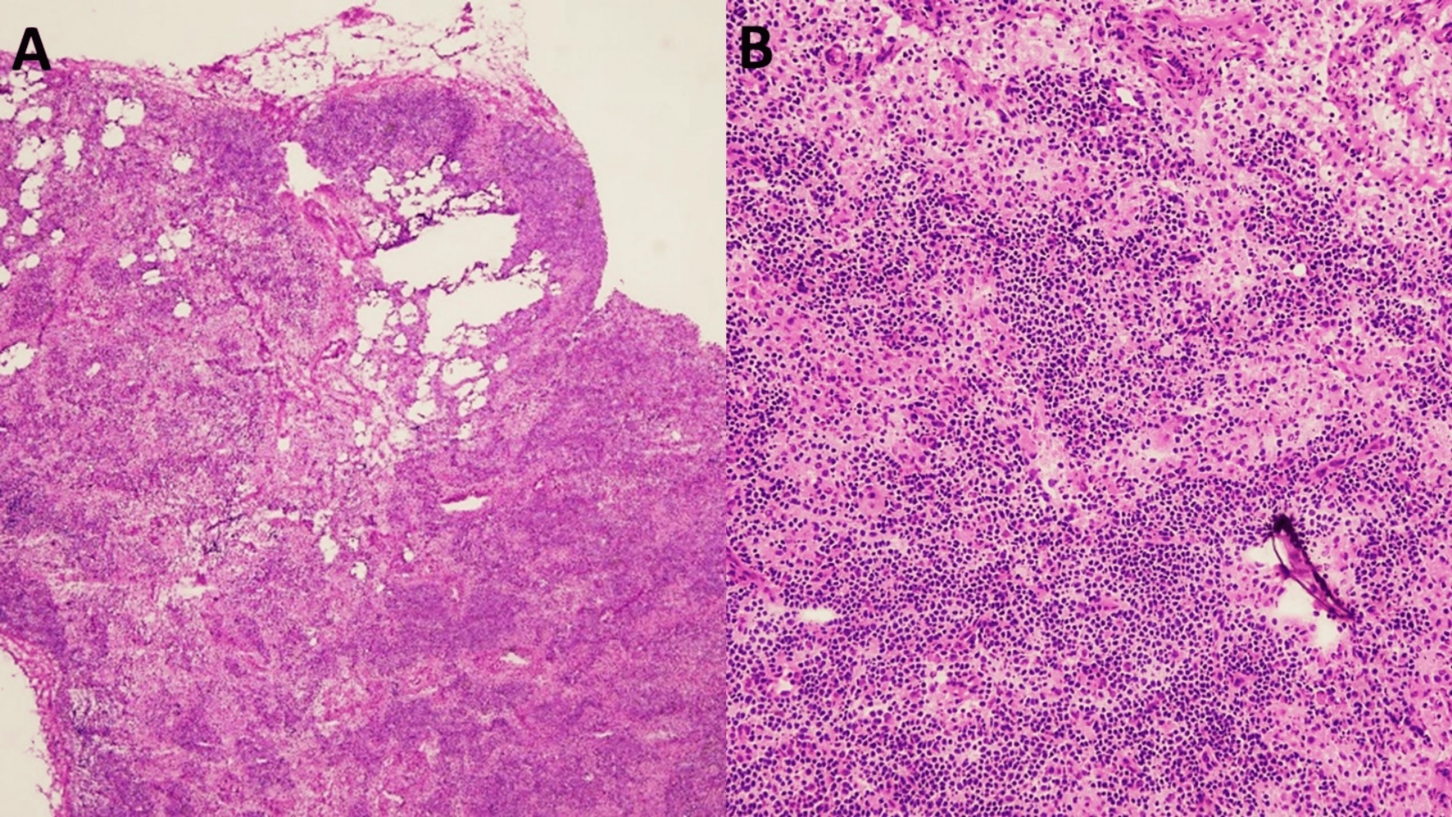
(A) Frozen section showing cutting artifacts (H&E, x20). (B) On higher magnification, treatment-related changes, including foamy macrophages, were identified (H&E, x40). H&E: hematoxylin and eosin.

**Figure 2:**
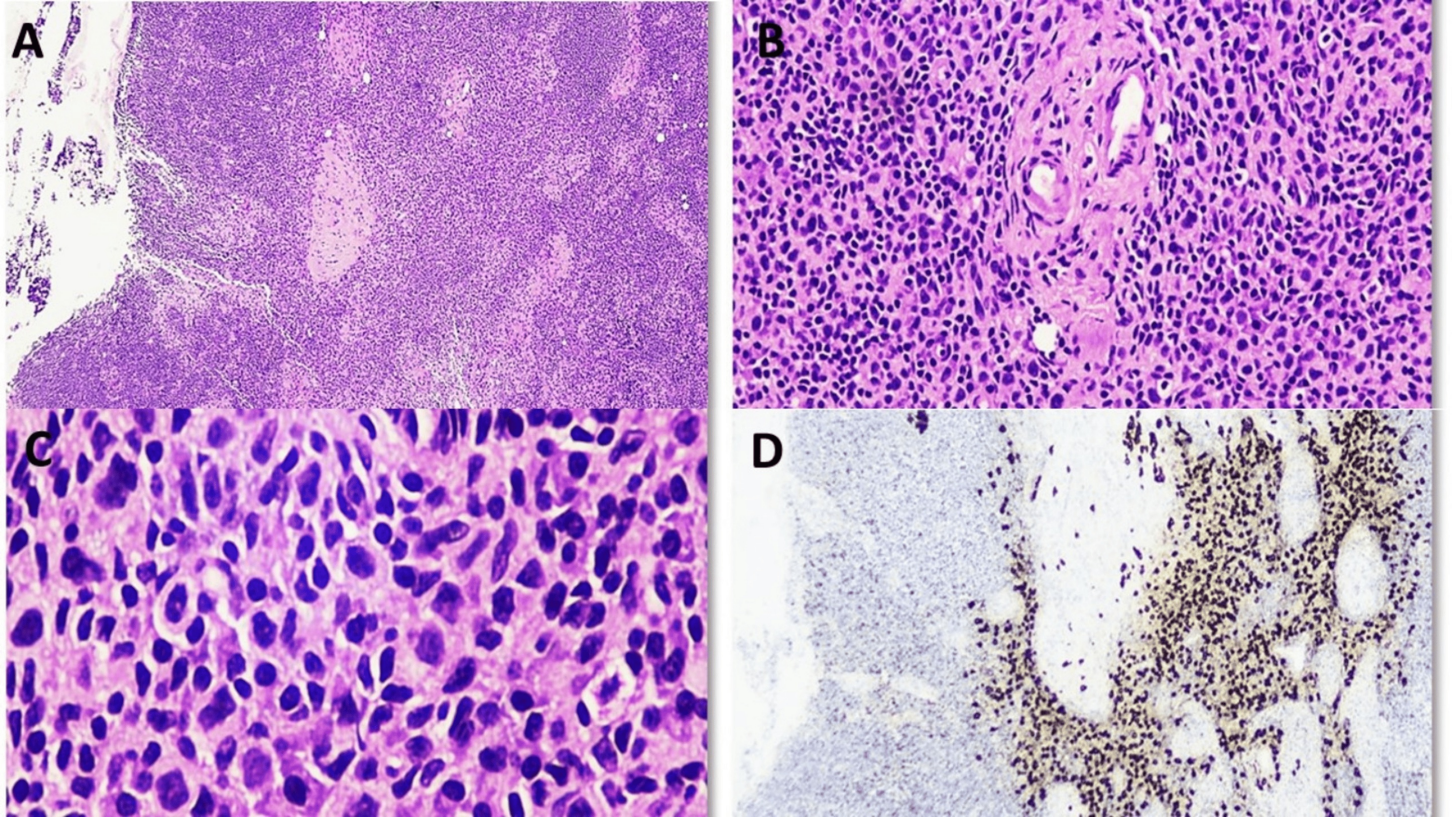
(A) Permanent section exhibiting the intact architecture of sentinel lymph node (H&E, x20). (B & C) Scattered tumor cells were identified with surrounding foamy macrophages and histiocytes (H&E, x40). (D) Cytokeratin AE1/AE3 highlighting tumor cells (H&E, x20). H&E: hematoxylin and eosin.

The sensitivity, specificity, and accuracy of frozen section analysis of SLNB were 83.00%, 100%, and 96.15% respectively. The FNR was 16.7% in our study [15]. The tissue was fixed in formalin and processed afterward for permanent sections. Out of 229 cases labeled as negative at the time of the frozen section, 220 (76.9%) cases were true negative confirmed on permanent sections. The remaining nine false negative cases and two deferred cases (as described in Table 2), in addition to 55 cases labeled as positive on the frozen section revealed small foci of tumor cells on permanent paraffin-embedded sections, and immunohistochemical stain cytokeratin AE1/AE3 was performed on all cases of invasive lobular carcinoma and sentinel lymph nodes exhibiting extensive therapy-related changes (as shown in Figures 1, 2). Total true positive cases on permanent sections were 66 (23.1%). The mean size of the nodal deposit of 11 discrepant cases was 0.2 cm (range = 0.02 to 0.6 cm).

**Table 2:** Summary of cases that were false negative and deferred at the time of the frozen section (n = 11).

Parameters	Result
Total number	N = 11
Patient age (years)	46 (30-55)
Diagnosis	
Invasive ductal carcinoma	9 (81.8%)
Invasive lobular carcinoma	2 (18.2%)
Histological grade before treatment	
Grade 2	9 (81.8%)
Grade 3	2 (18.2%)
Total number of sentinel lymph nodes received	3 (2-11)
Size of largest nodal deposit on permanent sections (cm)	0.2 (0.02-0.6)

**Figure 3:**
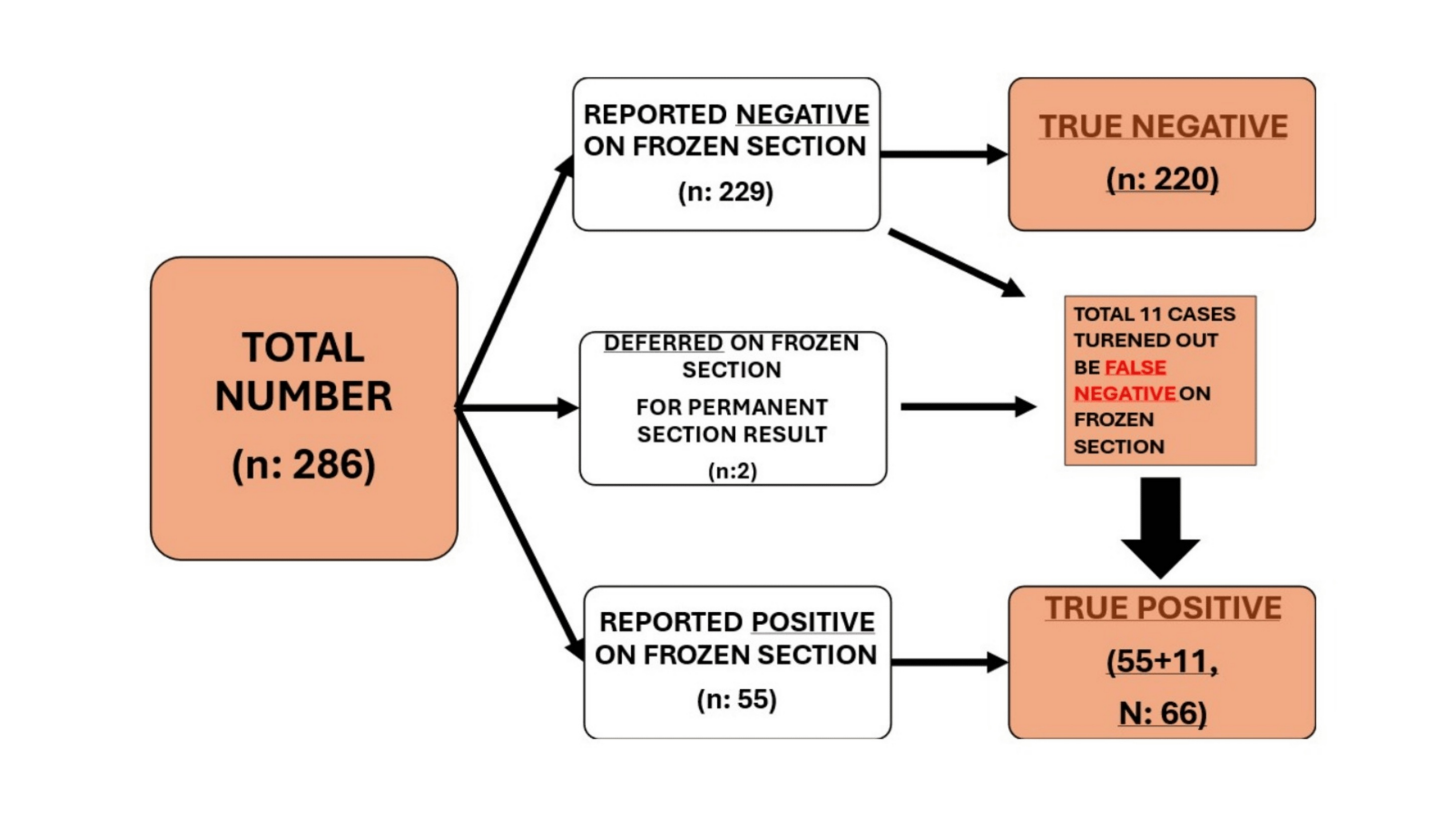
Brief overview of overall results of the study.

The summary of the overall results is shown in Figure 3.

The sensitivity, specificity, and accuracy of frozen section analysis of SLNB were 83.00%, 100%, and 96.15%, respectively (as shown in Tables 3, 4). The FNR was 16.7% in our study, which is within the range, as reported in earlier studies to be between 5.5% and 43% [15].

**Table 3:** Evaluation of accuracy of frozen section (n = 286). SLNB: sentinel lymph node biopsy.

	SLNB permanent section result			
Sentinel lymph node biopsy frozen section result	Present	N	Absent	N	Total
Positive	True positive	a = 55	False positive	c = 0	a + c = 55
Negative	False negative	b = 11	True negative	d = 220	b + d = 231
Total		a + b = 66		c + d = 220	286

**Table 4:** Descriptive statistical analysis details of frozen and permanent sections results: a tertiary care hospital experience.

Statistics	Value	95% CI
Sensitivity	83.33%	72.13% to 91.38%
Specificity	100.00%	98.34% to 100.00%
Positive predictive value	100.00%	93.51% to 100.00%
Negative predictive value	95.24%	92.10% to 97.17%
Accuracy	96.15%	93.22% to 98.06%

## Discussion

The use of SLNB has increased significantly during the past years to avoid complications associated with ALND. SLNB in post-NACT settings has a tremendous impact on axillary management in both mastectomy and breast-conserving settings. The sensitivity, predictive values, and FNR of SLNB in post-NACT settings are heavily reliant on multidisciplinary collaboration among breast surgeons and histopathologists. Correct identification of sentinel lymph nodes by surgeons and detailed evaluation by experienced pathologists can lower the FNR. Interpretation in post-NACT settings at times can be challenging for pathologists due to the presence of extensive therapy-related changes and fat replacement. FNR of SLNB demands performing level II ALND, requiring another hospital admission and procedure [12,16].

The tumor response is often heterogeneous, and evaluating SLNB in post-NACT can be challenging for the pathologist at times. This is because, in contrast to pre-treatment settings, the tumor response, which causes fibrosis, multinucleated giant cells, and collections of foamy macrophages, may mimic metastatic deposits, and increase FNR. To avoid false-positive diagnoses and unnecessary ALND, the threshold for deferral of the final diagnosis to a permanent section should be reduced. The deferred cases are then evaluated on permanent sections, and where necessary ancillary studies, including immunohistochemical stains, are used for confirmation [8].

We present the largest data about the sensitivity and predictive values of frozen sections of SLNB in a post-NACT setting from a tertiary care hospital in a resource-limited country. The results of intraoperative frozen section analysis with permanent sections in patients treated with post-NACT were evaluated and compared. There were no false positive cases in our study, but the FNR was 16.7%, which is within the range as reported in earlier studies between 5.5% and 43%. It is impractical to presume that every metastatic deposit detected on permanent serial deeper sections or by immunohistochemistry stain will likewise be detected at the time of the frozen section [10-13].

The average number of sentinel lymph nodes received for the frozen section was three (range = 2-4) in our study. The size of a nodal deposit is an important predictor of axillary metastases and is important for deciding further treatment plans. In our study, the mean size of the SLNB nodal deposit was 6 mm (range = 3 to 11 mm) as compared to the mean size of 7 mm (range = 1 to 20 mm) reported in a study by Moatasim et al. (2013), including pre and post-NACT patients [16].

Several errors, including technical, sampling, and interpretation errors, are significant in the determination of FNR. The 8/11 false negative cases on frozen sections in our study were those that showed metastases with a mean size of 0.2 cm (range = 0.02-0.6 cm) on permanent sections and were due to extensive fat replacement of lymph nodes that interfered with adequate cutting on frozen sections. Technical issues may affect the accuracy of frozen section analysis. The correlation between the accuracy rate and the number of frozen section levels examined was addressed by Turner et al. [17]. While false negative predictive value and accuracy were increased at four frozen section levels in that investigation, the differences were not statistically significant. Four sections are routinely examined at the time of frozen section reporting in our setting. Increasing the sampling of nodal sections is an additional strategy to optimize sensitivity and accuracy. The "exhaustive intraoperative frozen section method" of detection with a better FNR was reported by Veronesi et al. [18]. FNR of 32.1% was reported from their previous assessment, which comprised three sections from half a lymph node. The more recent technique takes 30 to 60 sections from each lymph node and takes an average of 40 to 50 minutes. This method significantly lowered the FNR to 5.5%. Unfortunately, this may not be practical for most institutions due to increased workload and shortage of experienced staff [13].

A total of 2/11 patients were known cases of invasive lobular carcinoma. Lobular carcinoma exhibits discohesive cells that can mimic lymphocytes and histiocytes and are difficult to diagnose at the time of frozen section [11,19]. It can be reduced significantly by the use of rapid immunohistochemistry at the time of frozen section but is not currently available in our setting.

One of the 11 false negative cases showed an interpretation error. One of the greatest challenges is to detect scattered metastatic deposits in extensive SLNB evaluation. To ensure the SLNB section's quality and consistent reporting when frozen sections are reported by multiple pathologists with interests in various sub-specialties on a rotating basis, it is recommended that a senior pathologist with experience in breast pathology should review at regular intervals [16,20].

In our study, the sensitivity, specificity, accuracy, positive predictive value, and negative predictive value were 83.33%, 100%, 96.15%, 100%, and 95.24%, respectively. In contrast, the study by Moatasim et al. (2013) included pre- and post-NACT patients, and reported sensitivity, specificity, accuracy, positive predictive value, and negative predictive values of intraoperative sentinel lymph node assessment as 90%, 100%, and 96%, 100%, and 94.1%, respectively, from our region [16].

A 2006 study from our institution looked at a total of 356 frozen sections, including all specimens performed during one year with deferral, discordant, and concordant rates of 3.93%, 2.92%, and 97.08%, respectively, with positive predictive values of 98.52% [20]. Compared with this study, the deferral rate of our department has significantly reduced to 0.7%, with a positive predictive value of 100%.

In the future, automated molecular assays will be used to detect metastases in sentinel lymph nodes at the time of frozen section evaluation. Future research should focus on more reliable, cost-effective techniques for areas with limited resources [19-21].

Our study had limitations, including a single institutional study with a small sample size and limited follow-up in the cases of false negative results. Further follow-up studies on patients with false negative results to know whether complete axillary dissection or additional therapy, including radiotherapy, were offered to them, and their prognosis rate as compared to patients who were reported as true negative, should be conducted.

## Conclusions

More research is needed to evaluate treatment options and follow-up in cases of discrepancy, sensitivity, and FNR of SLNB in post-NACT settings, including in countries with limited resources, larger sample sizes, and multi-center studies. The findings of this study will contribute to the limited regional database available for future studies.

Notwithstanding its constraints, the intraoperative frozen section is a reliable method to analyze the presence of SLNB metastases in breast cancer patients treated with NACT. Continuous monitoring of the rate of false positives and false negatives of frozen sections on SLNB is essential as feedback for pathologists to reduce errors, reduce the number of deferrals, and improve frozen section diagnosis.
